# The Role of Copy Number Variants in Gene Co-Expression Patterns for Luminal B Breast Tumors

**DOI:** 10.3389/fgene.2022.806607

**Published:** 2022-04-01

**Authors:** Candelario Hernández-Gómez, Enrique Hernández-Lemus, Jesús Espinal-Enríquez

**Affiliations:** ^1^ Computational Genomics Division, National Institute of Genomic Medicine, Mexico City, Mexico; ^2^ Centro de Ciencias de la Complejidad, Universidad Nacional Autónoma de México, Mexico City, Mexico

**Keywords:** breast cancer, co-expression networks, conditional mutual information, copy number variants, luminal B breast cancer

## Abstract

Gene co-expression networks have become a usual approach to integrate the vast amounts of information coming from gene expression studies in cancer cohorts. The reprogramming of the gene regulatory control and the molecular pathways depending on such control are central to the characterization of the disease, aiming to unveil the consequences for cancer prognosis and therapeutics. There is, however, a multitude of factors which have been associated with anomalous control of gene expression in cancer. In the particular case of co-expression patterns, we have previously documented a phenomenon of loss of long distance co-expression in several cancer types, including breast cancer. Of the many potential factors that may contribute to this phenomenology, copy number variants (CNVs) have been often discussed. However, no systematic assessment of the role that CNVs may play in shaping gene co-expression patterns in breast cancer has been performed to date. For this reason we have decided to develop such analysis. In this study, we focus on using probabilistic modeling techniques to evaluate to what extent CNVs affect the phenomenon of long/short range co-expression in Luminal B breast tumors. We analyzed the co-expression patterns in chromosome 8, since it is known to be affected by amplifications/deletions during cancer development. We found that the CNVs pattern in chromosome 8 of Luminal B network does not alter the co-expression patterns significantly, which means that the co-expression program in this cancer phenotype is not determined by CNV structure. Additionally, we found that region 8q24.3 is highly dense in interactions, as well as region p21.3. The most connected genes in this network belong to those cytobands and are associated with several manifestations of cancer in different tissues. Interestingly, among the most connected genes, we found MAF1 and POLR3D, which may constitute an axis of regulation of gene transcription, in particular for non-coding RNA species. We believe that by advancing on our knowledge of the molecular mechanisms behind gene regulation in cancer, we will be better equipped, not only to understand tumor biology, but also to broaden the scope of diagnostic, prognostic and therapeutic interventions to ultimately benefit oncologic patients.

## 1 Introduction

It is difficult to exaggerate the negative impact that cancer has, not only as a global health burden, but also as a phenomenon with enormous societal and economic consequences. Last year, cancer was the cause of death for 10 million people, mainly in poor and developing countries. Of all tumor types, breast cancer is the malignant neoplasm with the largest incidence worldwide (Siegel et al., 2020). Many survivors of the disease (five or more years after diagnosis) still have to live with physical and psychological problems that persist over time (Stein et al., 2008) and drastically reduce their quality of life and productivity.

Breast cancer is also a highly complex and heterogeneous disease, both from the molecular and from the clinical/phenotypic standpoints. It is known that breast cancer diagnosis, response to treatment, relapse, and outcome are largely dependent on the molecular features that have been associated with the so-called breast cancer subtypes (Liu et al., 2007; Kittaneh et al., 2013; de Anda-Jáuregui et al., 2016): Luminal A, the most common, estrogen and progesterone receptor positive but, in general, epidermal growth factor 2 receptor negative; Luminal B subtype is positive for estrogen receptor and epidermal growth and negative for progesterone; HER2+, these tumors are negative for estrogen and progesterone and positive for epidermal growth factor 2; finally the Basal subtype tumors, which are mostly (around 80%) *triple negative*, i.e., negative for estrogen, progesterone, and epidermal growth factor 2. These classifications are useful to determine the origin, evolution and treatment to be followed in each case, although each patient is unique and each subtype has peculiarities.

Of the estrogen-positive subtypes, Luminal B is often the most aggressive and although luminal breast tumors are susceptible to be treated with targeted therapy (a fact that commonly, but not always, is associated with better outcomes), in some cases present the worst prognosis for patients. This is so, due to several molecular and functional features that have been related to higher proliferation rates and pharmacological resistance (Cheang et al., 2009; Tran and Bedard, 2011; Creighton, 2012; Ades et al., 2014), or even due to metabolic alterations (Serrano-Carbajal et al., 2020).

Luminal B breast cancer subtype is hormone-receptor positive (estrogen-receptor and/or progesterone-receptor positive), and either HER2 positive or HER2 negative. This subtype presents high levels of Ki-67. In general, Luminal B tumors grow slightly faster than those from Luminal A subtype. Additionally, the prognosis is commonly worse (Li et al., 2016).

Luminal B breast tumors are characterized by a lower expression of estrogen receptor, and low expression of progesterone receptor (Harbeck et al., 2013). It is also defined by aggressive clinical behavior; its prognosis is similar to that of non-luminal cancers (Tran and Bedard, 2011). Bone metastasis appears more often in Luminal B patients than in non-luminal ones. However, recurrence or metastasis in this subtype have a better prognosis after treatment than non-luminal tumors. It has also been shown that Luminal B subtype presents high metabolic deregulation (Li et al., 2016; Serrano-Carbajal et al., 2020). Luminal B subtype tumors accounted for nearly 40% of all breast cancers (Metzger-Filho et al., 2013). Therefore, understanding the molecular basis of the luminal B subtype is a matter of current concern.

One of the most actively investigated genomic regions in the manifestation of breast cancer is cytoband 8q24.3 (Wokolorczyk et al., 2008; Dorantes-Gilardi et al., 2021). This region results particularly relevant to study in the present context, since 8q24 has been repeatedly reported to harbor multiple variants associated with the incidence of breast cancer and other type of neoplasms (Tong et al., 2020). Indeed, genomic variants in 8q24 have been ascertained to be associated with risk of breast cancer on systematic reviews and meta-analyses (Wang et al., 2020). Particularly interesting is the fact that larger genomic alterations (including copy number variants, CNVs) have been linked to breast cancer onset (Jia et al., 2019), often via disruption of healthy breast cells transcriptional programmes (Ibragimova et al., 2020). Indeed, such effects have been actually linked via clinical and pathological features to breast tumors of the Luminal B subtype (or related: ER+, PR- and HER2-) giving rise to basal-like and endocrine resistant phenotypes (Liu et al., 2018).

It has been argued that some of these genomic alterations have relevant consequences for transcriptional regulation anomalies associated with cancer. Gene regulatory programmes are known to be altered, a fact that has been linked with the onset and development of tumor phenotypes (de Anda-Jáuregui et al., 2016; Hernández-Lemus et al., 2019). In this regard, our group has thoroughly described how the more relevant gene-gene co-expression interactions in several cancer types often occur between genes from the same chromosome (*cis-*), even in proximal chromosomal locations. Conversely, inter-chromosome (*trans-*) interactions (or even long distance intra-chromosome co-expression interactions) are comparatively less abundant and have lower values of diverse statistical dependency measures (Espinal-Enríquez et al., 2017; de Anda-Jáuregui et al., 2019; Dorantes-Gilardi et al., 2020; García-Cortés et al., 2020; Zamora-Fuentes et al., 2020; Andonegui-Elguera et al., 2021; Dorantes-Gilardi et al., 2021; García-Cortés et al., 2021).

Since a large number of molecular players and processes are known to be involved in (normal and) anomalous transcriptional regulatory patterns, establishing which, among the multitude of potential causes of this phenomenon in tumors are actually more relevant given the available experimental evidence becomes desirable. Particularly important has been the discussion on the effects that CNVs have on gene expression patterns in several diseases (Stranger et al., 2007; Henrichsen et al., 2009), including breast cancer (Kumaran et al., 2017; Ohshima et al., 2017; Safonov et al., 2017; Sun et al., 2018; Shao et al., 2019).

With this in mind, we have decided to investigate the effect that CNVs may have on the phenomenon of distance-associated gene co-expression in breast cancer. For the reasons already discussed we have chosen to perform a detailed study centered in the chromosome 8 and the 8q24 region in Luminal B breast tumors. In this study, we implemented a probabilistic modeling approach to evaluate how CNVs affect long and short range co-expression profiles in Luminal B breast tumors. We analyzed the co-expression patterns in chromosome 8, since it is known that amplifications and deletions in that chromosome may influence cancer development. We found that CNV signatures in chromosome 8 of Luminal B network do not significantly alter the co-expression patterns, hence the co-expression program in this tumor phenotype is not determined by CNV structure. Additionally, we found that the 8q24.3 region is highly dense in co-expression interactions, as well as p21.3. The most connected genes in this network belong to those cytobands and have been associated with several tumor types. Interestingly, among the most connected genes, we found MAF1 and POLR3D, which may constitute an axis of regulation of gene transcription, in particular for non-coding RNA species. We believe that by advancing our knowledge of the molecular mechanisms behind gene regulation in cancer, we will be improving, not only to understand tumor biology, but also the scope of diagnostic, prognostic and therapeutic interventions to ultimately benefit oncologic patients.

This article is organized as follows: Section 2 explains what conditional mutual information (CMI) is and why it is appropriate in the present investigation; the Kolmogorov-Smirnov method is also presented to quantify the difference between two probability distributions. In section 3, the method used to build the networks is explained. It is also established (via conditional mutual information distributions), that CNVs are not significantly associated with the structural features (in particular distance-dependent co-expression patterns) of the analyzed co-expression networks. The results are analyzed in section 4. There, the genes with the highest connectivity as well as their possible relationship in the development of cancer are identified and discussed. Finally, in section 5 the conclusions and some consequences of this work are presented.

## 2 Methods

### 2.1 Data Acquisition

The complete collection of The Cancer Genome Atlas (TCGA) breast RNA-Seq samples was downloaded in January 2019 from the GDC repository https://portal.gdc.cancer.gov/repository. This collection included 113 solid tissue, normal samples and 1,102 primary tumor samples. From these samples, 192 corresponded to Luminal B tumors. Data acquisition was carried out by using the TCGABiolinks R package (Colaprico et al., 2016).

### 2.2 Data Integration

An integrity check was carried out in raw expression files using gene annotations from BioMart. Only protein coding genes belonging to conventional chromosomes (1, 2, … , 22, X and Y) were kept. The CNVs of the micro-RNAs were *masked* from the 8q24.3 region and excluded from the analysis. Supplementary Materials S1, S2 contains gene expression and genetic information of chromosome 8 genes. Pre-processing and quality control of the gene expression samples were performed as in (Espinal-Enríquez et al., 2017). In brief, we used for NOIseq R library for quality control (Tarazona et al., 2011, 2015). For batch effect removal, normalization, transcript length and GC content correction, EDASeq library was implemented (Risso et al., 2011). Finally, for multi-dimensional noise reduction we used ARSyN R library (Nueda et al., 2012).

### 2.3 Conditional Mutual Information Measures

We considered information theoretical measures of statistical dependency as follows: Let *X*, *Y* and *Z* denote discrete random variables having the following features:

1 Finite alphabets X , Y and Z , respectively
2 Joint probability mass distributions *p* (*X*, *Y*, *Z*), and partial-joint probability mass distributions *p* (*X*, *Y*), *p* (*X*, *Z*), etc.,
3 Marginal probability mass distributions p (X), p (Y) and p (Z)

Let also $\hat{X}$ , $\hat{Y}$ and $\hat{Z}$ denote additional discrete random variables defined on *X*, *Y* and *Z* respectively, the associated probability mass distributions will be $\hat{p\left(X\right)}$ , $\hat{p\left(Y\right)}$ and $\hat{p\left(Z\right)}$ , their joint probability mass distribution $\hat{p\left(X,Y,Z\right)}$ defined on J , the joint probability sampling space; J=X×Y×Z . For particular realizations, we have *p*(*x*) = *P* (*X* = *x*), $\hat{p\left(y\right)}=P\left(\hat{Y}=y\right)$ , etc., It is possible to define the Conditional Mutual Information (CMI) function *I* (*X*; *Y*|*Z*) as follows:

$$I\left(X;Y|Z\right)=\underset{z\in Z}{\sum }\underset{y\in Y}{\sum }\underset{x\in X}{\sum }p_{X,Y,Z}\left(x,y,z\right)\log\frac{p_{Z}\left(z\right)p_{X,Y,Z}\left(x,y,z\right)}{p_{X,Z}\left(x,z\right)p_{Y,Z}\left(y,z\right)}$$ (1)

Formally *I* (*X*; *Y*|*Z*) is a measure representing the expected value of the mutual information of two random variables *X* and *Y* given the value of a third random value *Z*. Thus *I* (*X*; *Y*|*Z*) represents the expected value (w.r.t. *Z*) of the Kullback-Liebler divergence from the conditional joint distribution *P* (*X*, *Y*|*Z*) to the product of the conditional marginals *P* (*X*|*Z*) and *P* (*Y*|*Z*).

CMI calculations were performed with R infotheo library (Meyer and Meyer, 2009).

### 2.4 Assessment of the Impact of Copy Number Variant in Gene Co-Expression Programs

In order to ascertain to what extent the CNVs are able to influence the gene co-expression programs, we performed Kolmogorov-Smirnov (KS) tests to evaluate the differences among the diverse CMI distributions. The KS statistic between 2 distributions is defined as:

$$D_{n,m}=\sup_{x}|F_{1,n}\left(x\right)-F_{2,m}\left(x\right)|$$ (2)

here, *F*_1,*n*_ (*x*) and *F*_2,*m*_ (*x*) are the empirical distribution functions of the first and the second sets. Statistical significance of the KS tests is asymptotically given as follows.

The null hypothesis is rejected (at significance level *α*), whenever

$$D_{n,m}>c\left(\alpha \right)\sqrt{\frac{n+m}{n\cdot m}}$$ (3)

with $c\left(\alpha \right)=\sqrt{-\ln\left(\frac{\alpha }{2}\right)\cdot \frac{1}{2}}$

KS tests were performed with the ks. test library in R.

Circos plots visualizations were made using the BioCircos JavaScript library (Cui et al., 2016).

## 3 Implementation

It has been argued that mutual information (MI) is a reliable measure to establish links between genes in co-expression networks (Margolin et al., 2006a,b; Hernández-Lemus and Siqueiros-García, 2013; Lachmann et al., 2016). Given that there is generally a sufficient amount of data available to reconstruct the probability distributions associated with the expression of genes, it is possible to use it without major restrictions when measuring the statistical dependency structures between them (Hernández-Lemus and Rangel-Escareño, 2011). It is also a measure that takes into account the non-linear contributions of interdependence in the series, which makes it more appropriate in the context of the complex regulatory patterns of gene expression.

The adoption of MI-based network deconvolution methods has opened the entrance of the tenets of information theory in the analysis of biomolecular networks. However, when, as in the present work, it is necessary to evaluate the mutual information between two random variables (corresponding to each of the individual gene expression profiles in the present case), given a third, potentially influential feature, one relevant alternative is the evaluation of the Conditional Mutual Information measures. Indeed CMI has already been used in the construction of regulatory networks in a different but related context to the one presented here (Liang and Wang, 2008; Zhang et al., 2012).

An underlying problem when applying MI and CMI to establish the dependency between variables is choosing the method for reconstruction of the associated probability distributions. There are two main ways to do this, using the k-nearest neighbor non-parametric method (Kraskov et al., 2004) and through kernel density estimation (Terrell and Scott, 1992). In this work we have chosen the second method, in particular with a Gaussian kernel estimation. The implementation used is the one corresponding to the *R* package Infotheo.

The role of CNVs has been pointed out as a possible element causing the loss of co-expression with the distance that is observed in cancer and that has been reported previously (García-Cortés et al., 2021). Ideally, a non-cancerous person has two copies of each gene, however many disorders of genetic origin are caused by the deletion, repetition or insertion of DNA segments, sometimes as long as the arm of a chromosome or a complete chromosome. Given that in the past, they have been found to be responsible for genetic diseases, to think that they may also have a relevant role in the loss of co-expression is an appealing idea.

To systematically evaluate the contribution of CNVs in gene co-expression in cancer, it is convenient to use an area of the genome that is particularly active in its manifestation and to calculate the influence of CNVs both in that region and in the surrounding *areas*. As previously mentioned, 8q24.3 has been identified as particularly active and connected in co-expression networks, mainly in breast cancer tissues. Thus, we chose the Luminal B breast cancer subtype, given the poor prognosis compared with the Luminal A subtype. Furthermore, gene expression patterns have shown altered metabolic pathways even more evident than in Basal-like or HER2+ phenotypes (Serrano-Carbajal et al., 2020). We took the CNV values of 183 coding genes from the 8q24.3 region (Supplementary Materials S3, S4), we take them one by one as conditional and calculate the CMI of 442 genes, including not only those of this region but of the entire chromosome 8. Supplementary Materials S3–S6 show the copy number alteration map of all samples for chromosome 8.

We repeated the above calculation for the same genes with control tissue samples assuming a number of CNVs equal to two in all samples as conditional. From the obtained values we construct co-expression networks. For each of the 183 conditional layers there are 97,461 links. The question behind this analysis is: How important is the difference obtained between the different conditional layers? For control tissue networks the question becomes trivial, since all the conditionals are essentially the same, but for those corresponding to Luminal B this difference may be relevant due to the already documented anomalous CNV structure in the region.

**FIGURE 1:**
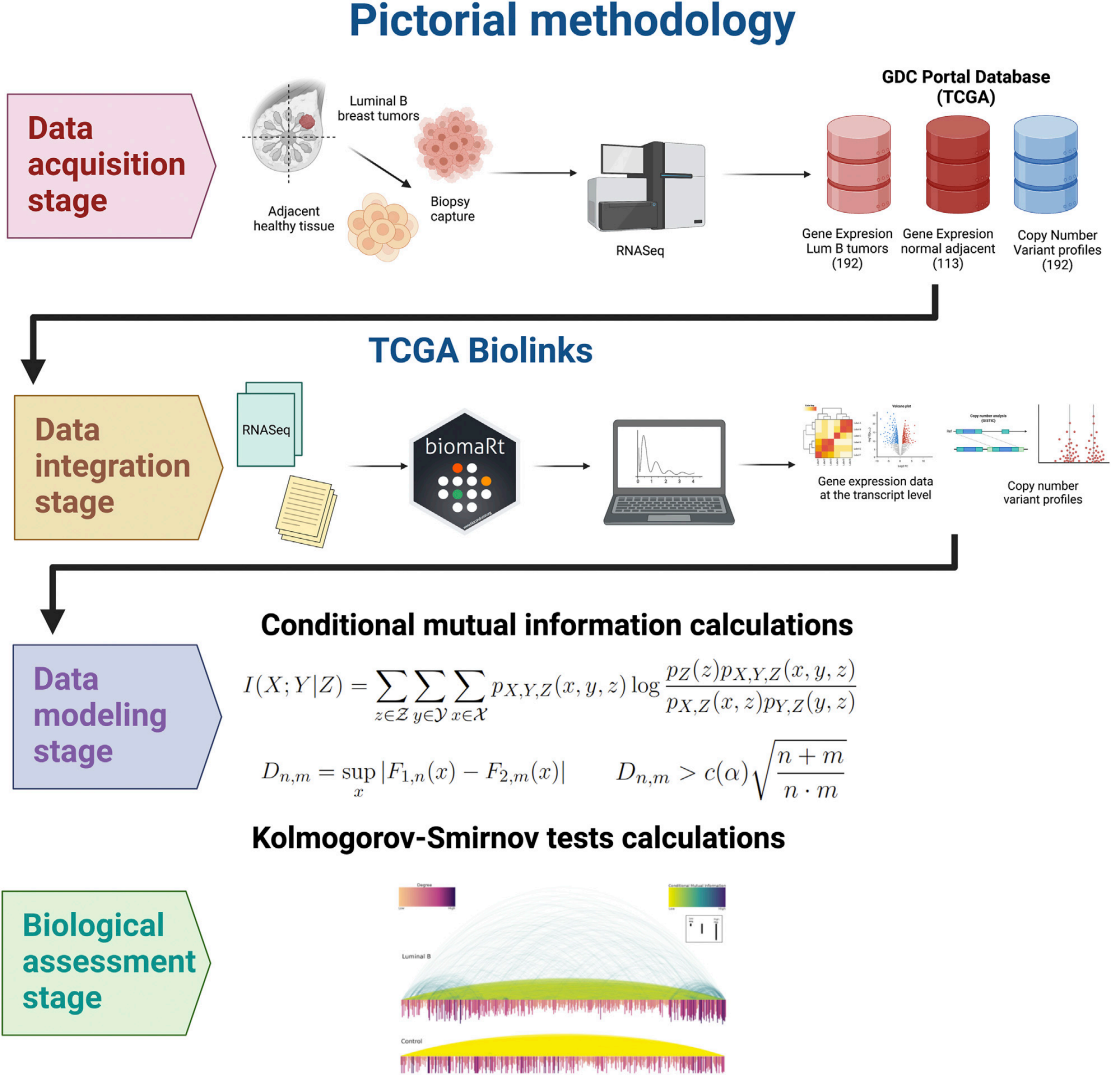
Workflow of this study. RNASeq obtained from tumor and control adjacent tissue biopsies (coming from the TCGA/GDC collaboration database) serves as a basis for conditional co-expression and CNV analysis. Samples were pre-processed for quality control and normalization, batch effect removal and mlti-level noise reduction. Copy number variation and RNA expression data were used to perform the conditional mutal information, Finally, spatial co-expression analysis was implemented.

To quantify the differences between each of the 183 conditional layers obtained, we calculated the difference between the distributions of CMI values for all possible pairs of them (16,653 comparisons) using the Kolmogorov-Smirnov test. In essence, this test compares the cumulative functions of two distributions and it establishes as a measure, the D statistic, i.e. the maximum vertical difference between them (Supplementary material S5). In Figure 1 we show graphically the workflow for this project.

## 4 Results and Discussion

### 4.1 Contribution of Copy Number Variants to the Co-Expression Program is Marginal

**FIGURE 2:**
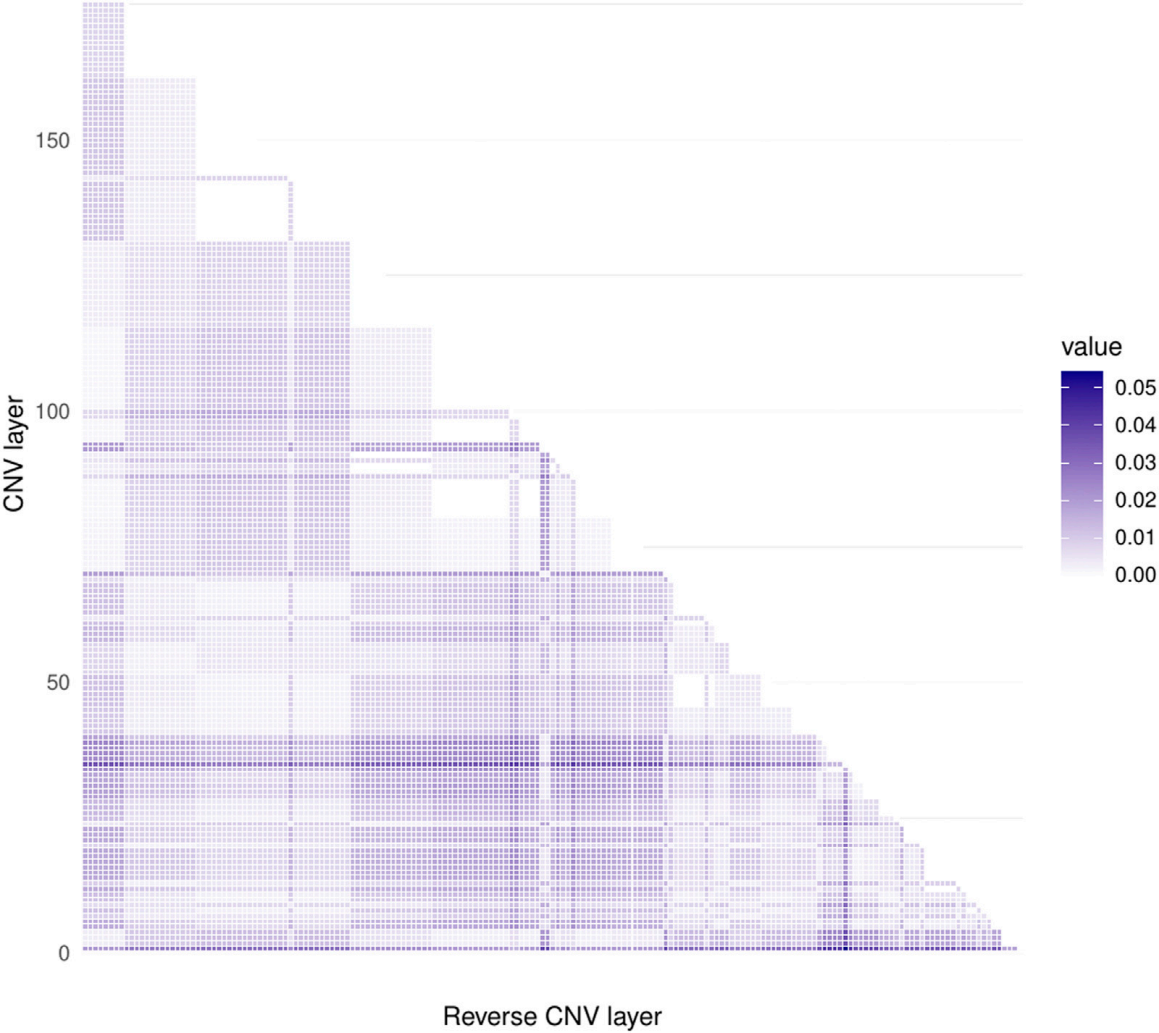
Heatmap corresponding to the values of the D statistic for the Kolmogorov-Smirnov test. Both axes represent the same CNV layers. As it can be observe from the color code at the right part of the figure (very low values of D), none of the CNV layers present a larger KS statistics, which reflects that copy number alterations do not significantly change gene co-expression values.

Figure 2 shows a heatmap with the values of the D statistic of the Kolmogorov-Smirnov test for each different pair of the 183 distributions of CMI values obtained. The largest of them, which occurs between the layers corresponding to the CNVs of the COL22A1 gene (ENSG00000169436) and (ENSG0000016943), is less than 0.06. This result indicates that the contribution of 8q24.3 CNVs in the co-expression networks of genes on chromosome 8 in the luminal B subtype of cancer is marginal.

This result acquires relevance since copy number alterations are a mesoscopic phenomena, which can affect large parts of the genome. On the other hand, changes in the co-expression landscape are considered a microscopic event, since those changes may affect specific genes and their regulatory relationships. Hence, with this result we provide evidence that a large-scale event such as amplifications/deletions of large portions of chromosome 8 do not significantly alter the gene co-expression program in cancer.

### 4.2 Conditional Mutual Information is Higher in Luminal B Than in Controls

**FIGURE 3:**
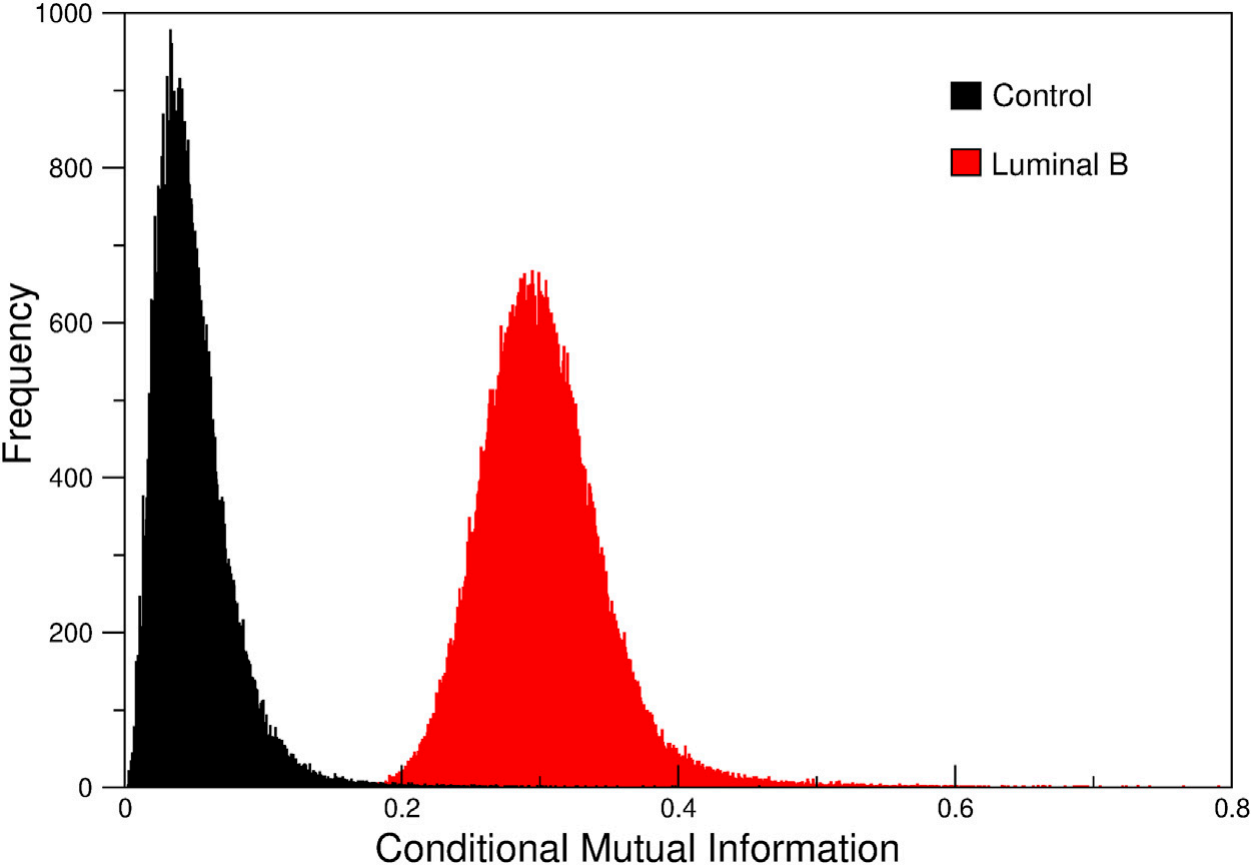
Conditional Mutual Information for normal adjacent tissue (black) and Luminal B tumors (red). We can notice that Luminal B tumors present distinctively higher values of CMI than Controls.

Since all the layers corresponding to Luminal B samples have essentially the same distribution of CMI, we take the first, whose conditionals are the CNVs of the COL22A1 gene, as representative of the entire family of networks and proceed to analyze it. Figure 3 shows the difference between CMI from the Luminal B subtype network and the one from the control network. As it can be clearly observed, the control CMI values are lower than the cancer counterpart.

In (Espinal-Enríquez et al., 2017), we observed that mutual information values from control gene co-expression networks are higher than in the cancer-derived network. However, the calculation was taken over all the gene-gene co-expression interactions, i.e. intra and inter-chromosomal gene correlations. In this case, the CMI is taken between chromosome 8 genes only, i.e., just intra-chromosome interactions.

Taking into account that the loss of long-range co-expression phenomenon has been previously reported in networks form different cancer types (Zamora-Fuentes et al., 2020; Andonegui-Elguera et al., 2021), and also for breast carcinoma (Espinal-Enríquez et al., 2017; Alcalá-Corona et al., 2018; de Anda-Jáuregui et al., 2019; Dorantes-Gilardi et al., 2020), and in particular for breast cancer subtype networks (de Anda-Jáuregui et al., 2019; García-Cortés et al., 2020; Dorantes-Gilardi et al., 2021), the finding of a higher average co-expression in intra-chromosome 8 of Luminal B breast cancer compared with the control one, reinforces the fact that this phenomenon is a common trait in several types of cancer.

### 4.3 Cancer Network *Grows* in Small Local Regions, but not in Control

**FIGURE 4:**
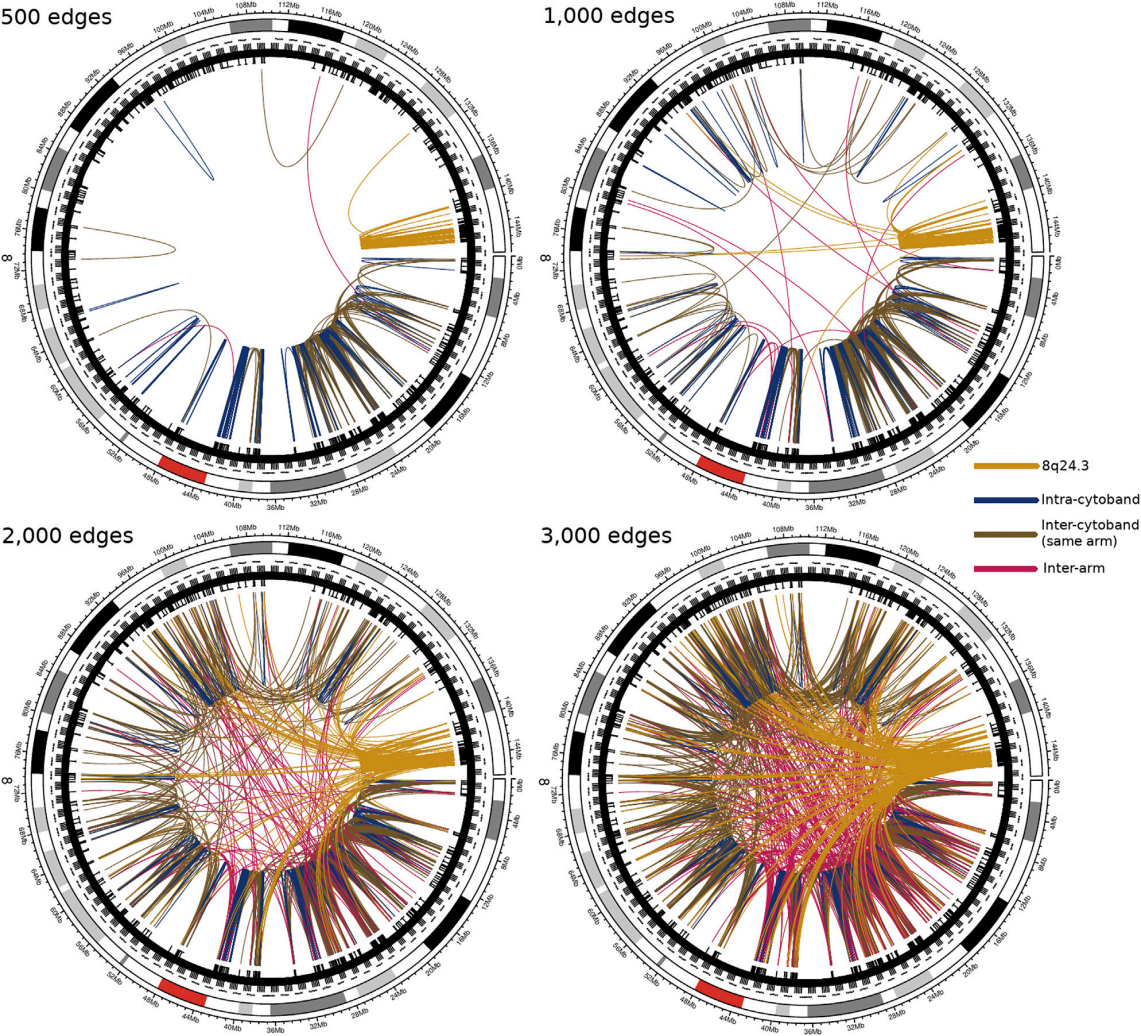
Top interactions of Luminal B CMI network at different cut-offs (500, 1,000, 2,000, and 3,000 edges). At first, the intra-cytoband interactions dominate, mainly in q24.3, p21.3, p11.21 and p11.23; secondly, the inter-cytoband interactions (particularly in p-arm), and finally, the inter-arm edges. Red arc at the external circle represents the centromere of Chr8. Color code of the co-expression interactions are also described. The take-home message is that for the top interactions, the location of participating genes is very close between them, in particular, at cytoband q24.3.

Figure 4 corresponds to Luminal B subtype interactions. Top-left to bottom-right shows the Top-500, 1,000, 2,000, and 3,000 interactions in chromosome 8. As it can be appreciated, interactions appear local in the first place, markedly in cytobands q24.3 (yellow arcs) and p11.23. Subsequently, inter-cytoband and inter-arm interactions occur, but in a small fraction (top-right). For the top-2,000 edges, it is clear that several interactions appear from q24.3. Finally, for the top-3,000 edges, 8q24.3 region is strongly connected with the rest of chromosome 8, and more inter-arm and inter-cytoband edges appear.

**FIGURE 5:**
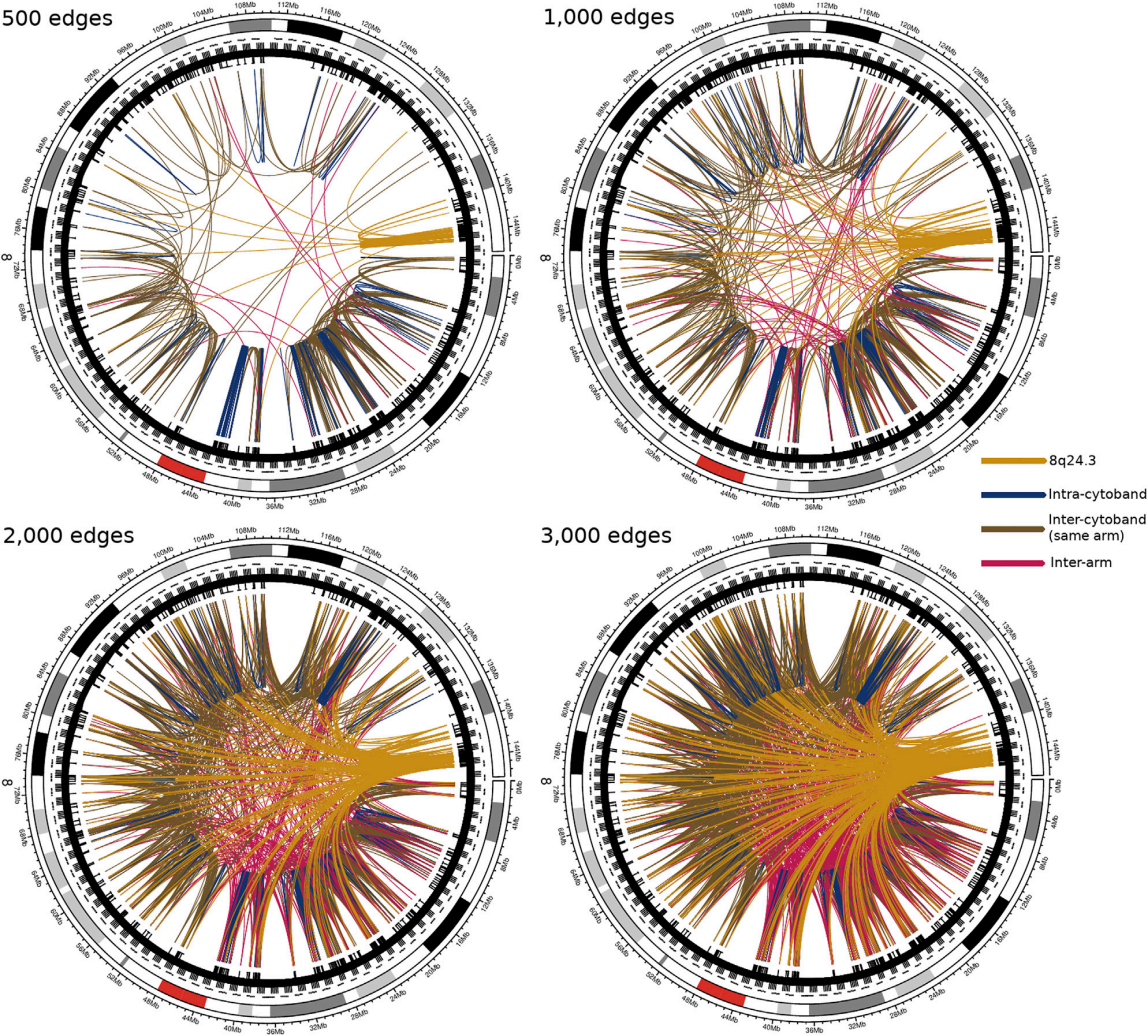
Top interactions of control tissue CMI network at different cut-offs. In this figure, analogue to Figure 4, although both, expression and co-expression are not uniform, many of the strongest links correspond to inter-arm interactions. The color code is the same than in Figure 4. It is clear the difference between the area covered by top-3,000 interactions in control circos-plot and the one observed in the Luminal B case. The density of links in the control network is larger than in Figure 4.

On the other hand, the Figure 5 shows the existence of a non-localized connectivity for the control network; since for the first 500 highest connections, interactions occur between genes from different cytobands, or even from different arms, much more often than in the cancer phenotype. The same effect is shown in the top-1,000 and 2,000 interaction circos plots. It is worth noticing the small number of intra-cytoband interactions, even in the top-3,000 edges (830 out of 3,000, dark blue edges).

We believe that this pattern where the highest correlations appear between physically close genes, is in agreement with other phenomena observed in breast and other cancers in which the loss of long-distance co-expression is evident, such as kidney (Zamora-Fuentes et al., 2020) or lung (Andonegui-Elguera et al., 2021).

### 4.4 Extreme Regions Exhibit a High Connectivity Pattern in Luminal B Network

**FIGURE 6:**
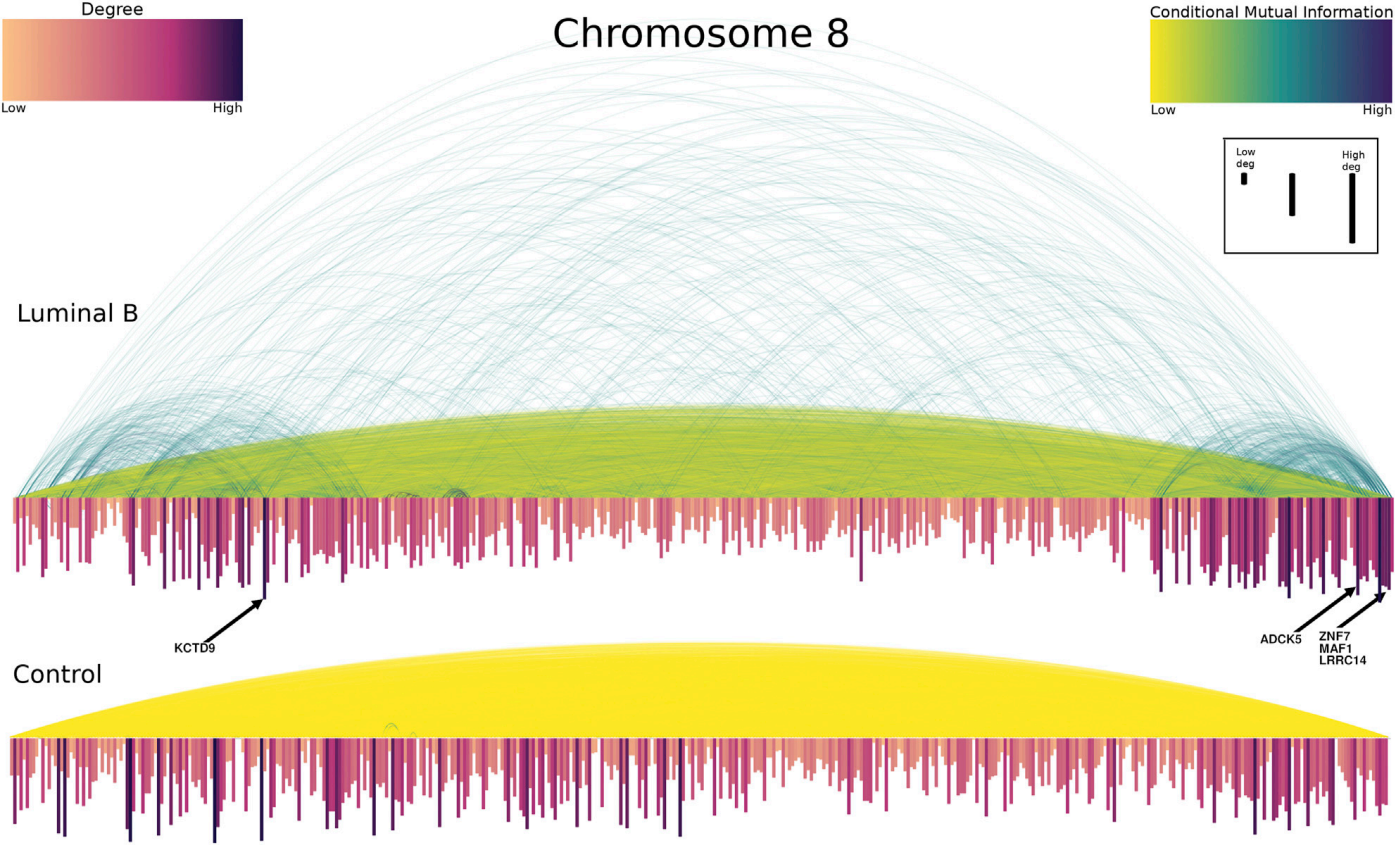
Top CMI values between genes of Chromosome 8 for Luminal B (upper) and control (lower) networks. In this figure, the genes are placed according to its start position. Yellow-to-blue arcs represent co-expression interactions between the connecting genes. The size and color of the genes are proportional to the degree. As it can be observed, for the case of Luminal B, the extreme left and right sides contain the majority of highest CMI interactions (dark arcs), in particular, cytobands q24.3 and p11.21–23. Conversely, in the control network, interactions are not particularly biased to a specific region. Top-degree genes are indicated with black arrows. ZNF7, MAF1 and LRRC14 genes are overlapped in the figure.

We decided to analyze the topology of the resulting networks, but we only kept the highest interactions for this purpose. We set 0.35 as a threshold for CMI in the Luminal B network. This threshold resulted in a network with 11,449 edges and 420 genes. For comparison, we conserved the same number of edges in the control network. Taking into account the highest CMI values in the Luminal B phenotype, a very intriguing phenomenon appears: the first (8p11.21–23) and last (8q24.3) codifying regions of the chromosome 8 exhibit strong connections. Additionally, this strength decrease towards the centromere. Conversely, in the control network, the strength of interactions does not depend on the location of genes. This remarkable difference between the breast cancer and the non-tumor adjacent phenotype can be appreciated in Figure 6

Regarding the Luminal B chromosome 8 network, the genes of the extreme regions also constitute the most connected nodes of the network. The case of cytoband q24.3 is the most emblematic one. Cytoband q24.3 is the one with more intra-cytoband edges (1,640 out of 11,449). It is also the cytoband with more genes in the network (79 out of 420). Regarding the inter-cytoband edges, the large majority of interactions from any cytoband also correspond to those from q24.3. That is the reason for which the average degree of region q24.3 is the highest of the network (52). The non-extreme region p11.23 is an exception that has strong interactions.

It is important to mention that in region p11.23, there are some crucial genes in terms of cell maintenance, as well as for cancer development. For instance, four genes, namely BAG4, LSM1, ASH2L and BRF2 serve as housekeeping genes. On the other hand, FGFR, a keystone gene in cancer development, has been observed to be amplified in luminal B breast tumors (Erber et al., 2020; Voutsadakis, 2020; Amina et al., 2021).

### 4.5 Highly Connected Genes and Their Possible Relationship With Cancer

In Table 1 we show the most connected genes in the Luminal B network. As it can be observed, most of the genes belong to region q24.3. However, three genes, KCTD9, POL3RD, and ATP6V1B2 belong to the region p21.3. The gene with the highest Betweenness Centrality (BC) is KCTD9. Below we will provide a brief summary of what it is known of those genes in Luminal B breast cancer or other carcinomas.

**TABLE 1:** The most connected genes belong to q24.3 and p21.2.

Gene	Band	Degree	BC	*Log*_2_ *FC*
ZNF7	q24.3	109	0.00544	0.47
KCTD9	p21.2	106	0.00656	−1.16
MAF1	q24.3	105	0.00562	0.07
LRRC14	q24.3	102	0.00512	0.34
ADCK5	q24.3	101	0.00523	0.93
PTK2	q24.3	99	0.00516	−0.03
POLR3D	p21.3	97	0.00508	−0.36
ZNF16	q24.3	97	0.00518	0.38
ATP6V1B2	p21.3	96	0.00579	−0.15
ZNF707	q24.3	96	0.00410	0.99

ZNF7 (Zinc Finger Protein 7) has been indicated as a biomarker of survival in glioblastoma Esteve-Codina et al. (2021) and Burkitt’s lymphoma (Gallego and Lazo, 1994). ZNF7 is the most connected gene in the Luminal B breast cancer co-expression network, and it is slighlty overexpressed (Table 1).

Many genes on chromosome 8 have been associated with mental illnesses, mainly schizophrenia, and their mutations are presumed to be involved in the development of our mental abilities. This is the case of the coding genes for the KCTD (Potassium Channel Tetramerization Domain) proteins. However, it has recently been indicated (Angrisani et al., 2021) that the 25 members of this family are potentially involved in a second fundamental activity: 13 of them have an exclusive pro-tumor function, 5 an exclusive anti-tumor function, 5 a pro/anti-tumor role, and 2 with a function not yet determined. KCTD9 has a pro-tumor function not yet reported in the literature, but inferred through databases. However, in this case, KCTD9 is underexpressed (−1.16) which may implicate a dual role in the phenotype.

MAF1 gene is known for its regulatory effect on the polymerase III, although it has also been associated with cancer, given that it activates the expression of the PTEN protein, which is an important tumor suppressor (Zhang et al., 2018). Interestingly POLR3D is also one of the most connected genes in the Luminal B breast cancer network. POLR3D gene encodes an RNA polymerase 3 subunit D, and synthesizes small RNAs, such as 5S rRNA and tRNAs, which, when inhibited by TRIPLIDE (TPL) influence the control of colorectal cancer (Liang et al., 2019). POLR3D is also inhibited by miR-320 (Ramassone et al., 2018).

The fact that MAF1 and POLR3D genes were highly connected could be an indicator of alterations in the transcriptional regulation program. Since MAF1 regulates polymerase III, and POLR3D encodes an RNA polymerase III subunit, the emergence of an axis of transcriptional regulation of non-coding RNA species results appealing. The latter may imply that in the Luminal B subtype, transcriptional regulation mediated by non-coding RNAs could affect the gene regulatory program. The latter coincides with the fact that POLR3D abnormal activity is characteristic of cancer cells (White, 2004).

ADCK5 has been indicated as an intermediary in the growth and metastasis of lung cancer. This gene promotes invasion and migration of lung cancer cells through the ADCK5-SOX9-PTTG1 pathway Qiu et al. (2020).

Regarding PTK2, this gene in association with KCNMA1 gene has been reported as a tumor suppressor in gastric cancer Ma et al. (2017). Additionally, the SMARCE1 gene regulates metastasis in breast cancer through its interaction with HIF1A and PTK2 Sethuraman et al. (2016). It has also been associated with hepatocarcinoma (Okamoto et al., 2003). Additionally, PTK2/FAK is considered a driver of radio-resistance in HPV-negative head and neck cancer (Skinner et al., 2016).

Recently, the FAM83H gene (also located on chromosome 8, specifically in the 8q24.3 region) has been related to Zinc Finger Proteins (ZNFs) genes, specifically ZNF16. Gallbladder cancer is highly associated with the expression of these two genes Ahn et al. (2020).

ATP6V1B2 gene has been identified as a possible biomarker (from controlled-to-aggressive growth with invasion of muscle tissue) for bladder cancer. Specifically, it is underexpressed in the early stages of the disease and over-expressed in the advanced stages Fang et al. (2013). Additionally, it has been associated with follicular lymphoma, activating autophagic flux and mTOR pathway (Wang et al., 2019).

ZNF707 has recently been Kim et al. (2020) identified as highly overexpressed in the Japanese population. High expression has also been shown in kidney and luminal B cancer patients Machnik et al. (2019) which suggests that ZNF707 could be involved in the development of cancer in general, regardless of the tissue.

In brief, we can establish that the most connected genes in the chromosome 8 network for Luminal B breast cancer are significantly related to the oncogenic phenotype. Particularly interesting is the case of MAF1, which in turn regulates POL3RD expression, suggesting an axis of non-coding RNA regulation.

As it can be also observed in Table 1, the differential expression in the large majority of hub genes is not significant. However, the co-expression patterns of these genes are importantly different than in the control case. We have observed previously, in clear cell renal carcinoma progression, that the gene expression patterns do not change significantly over progression stages, however, the co-expression networks are clearly different between those stages (Zamora-Fuentes et al., 2020). Therefore, slightly different gene expression patterns may implicate a dramatical alteration in the co-expression landscape. The differential expression of all Chr8 genes is provided in Supplementary Material S6.

## 5 Conclusion

The understanding of the intricate relationship between copy number alterations, which can be seen as a mesoscopic dimension, with the regulation of the gene co-expression program, which can be understood as a microscopic phenomenon, is a highly promissory pathway for intense research in the near future. In this work, we have shown, for the case of Chromosome 8 in Luminal B breast cancer, that the Copy Number Alteration scenario does not influence, in a relevant manner, the Conditional Mutual Information program. That is independent on which region is analyzed. As a summary of findings, we can establish the following:

• CNVs do not influence Conditional Mutual Information (as observed in Figure 2).
• Co-expression program in the chromosome 8 for Luminal B breast cancer shows localized hotspots regions in certain cytobands.
• The large majority of Chromosome 8 gene co-expression interactions shows low CMI values, meanwhile the extreme parts of the chromosome show higher values.
• Cytoband q24.3 has the highest values of MI, is the most dense in terms of interactions, and its genes have the highest degree.
• In the control phenotype there is a homogeneous CMI distribution regarding the location of genes in the top interactions, contrary to the case of Luminal B network.
• Taking into account a *growing* of the networks from highest to lowest CMI values, in the case of Luminal B network, the top CMI values appear between intra-cytoband genes; after that, between inter-cytoband and same-arm genes; finally between inter-arm genes. In the case of the control network, there is no clear localization pattern.
• Genes such as ZNF7, KCTD9, MAF1, or POLR3D have the highest degree centralities. Those genes have been reported to have influence in cancer.
• MAF1 and POLR3D could form an axis of non-coding RNA regulation, which can be a possible complex for future research.

Further steps towards a whole understanding of how copy number alterations may affect the co-expression program in breast cancer must include the analysis of the conditional mutual information of all chromosomes in Luminal B breast cancer. A similar study in the other molecular subtypes is also needed. The influence of the progression stage must be also taken into account. Finally, this analysis over other cancer tissues will provide a solid and robust landscape of the role of copy number alterations in the rise and development of cancer.

## Data Availability

The datasets presented in this study can be found in online repositories. The names of the repository/repositories and accession number(s) can be found in the article/Supplementary Material.

## Supplementary Material

Supplementary Material S1 Gene expression data. This table includes the normalized RNA-seq expression of all Luminal B samples.

Supplementary Material S2 Gene information. Name, ENSEMBL-id, GC content, biotype, gene description, cytoband location, base pair and base end of each gene of chromosome 8 is provided.

Supplementary Material S3 CNV data. This table contains the copy number alteration information for all Luminal B breast cancer samples.

Supplementary Material S4 Gene information data from copy number regions.

Supplementary Material S5–S8 Chromosome 8 copy number alteration map of 140 Luminal B samples. For these figures we used the IGV platform (https://software.broadinstitute.org/software/igv/).

Supplementary Material S9 D-statistic heatmap and p-values. In the first figure, the layers with the largest discrepancies, all less than 0.05, appear in red. The corresponding p-values appear in the second heatmap; these should be read in conjunction with the D-values. Most of these numbers are low and appear in shades of white, the blue dots correspond to *p*-values equal to one, indicating that the data are the same in both distributions. Like adjacency matrices, these matrices are symmetric.

Supplementary Material S10 Differential gene expression of Chr8. Log2 fold change and *p*-values are provided.
