# Supplementary figures and images for: The Role of Copy Number Variants in Gene Co-Expression Patterns for Luminal B Breast Tumors
Source: Front Genet. 2022 Apr 1;13:806607. doi: 10.3389/fgene.2022.806607 (PMC9010943; doi:10.3389/fgene.2022.806607)

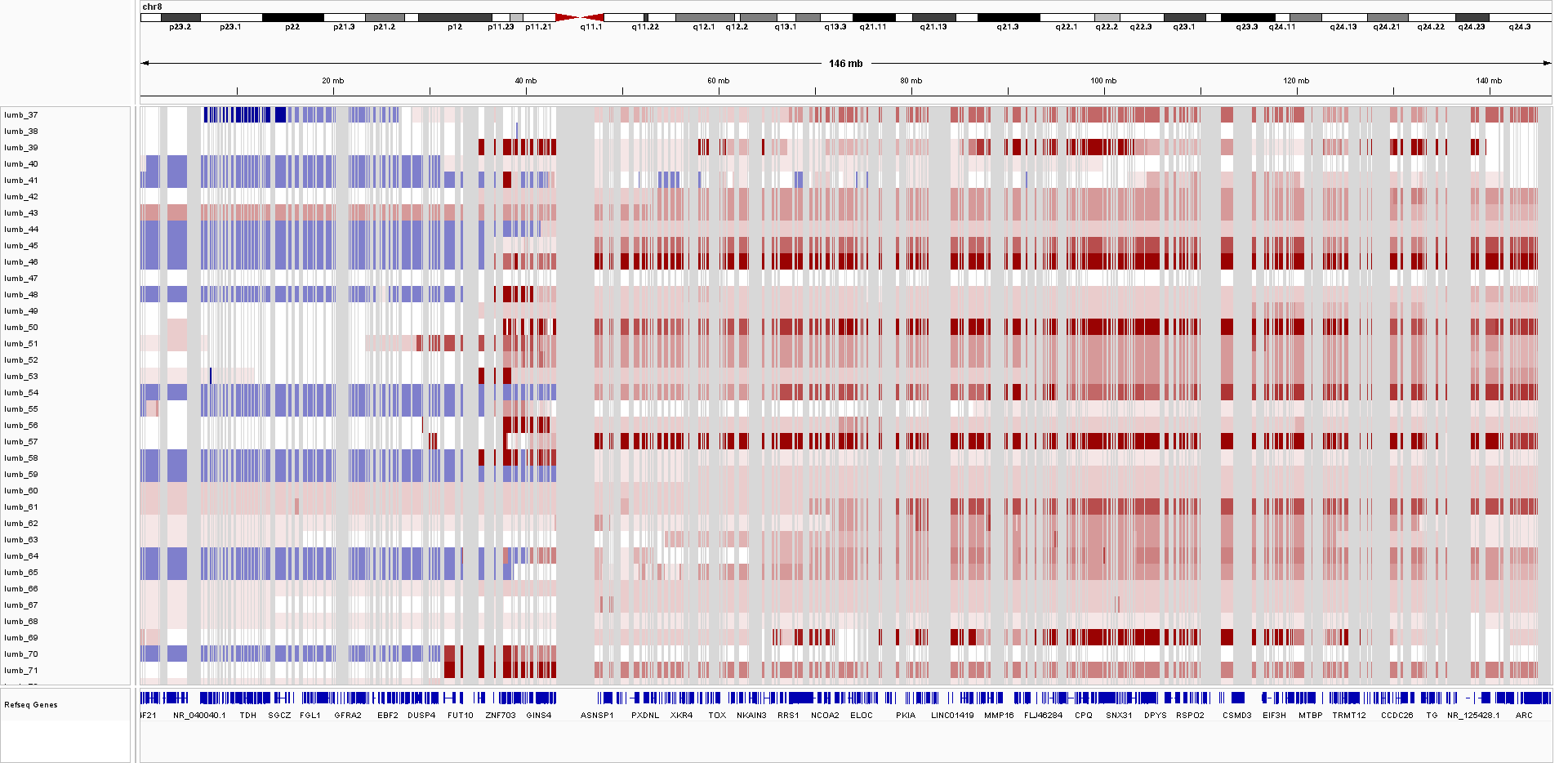

Supplement: Supplementary file 1 [file DataSheet1.zip › Supplementary/S6.png]

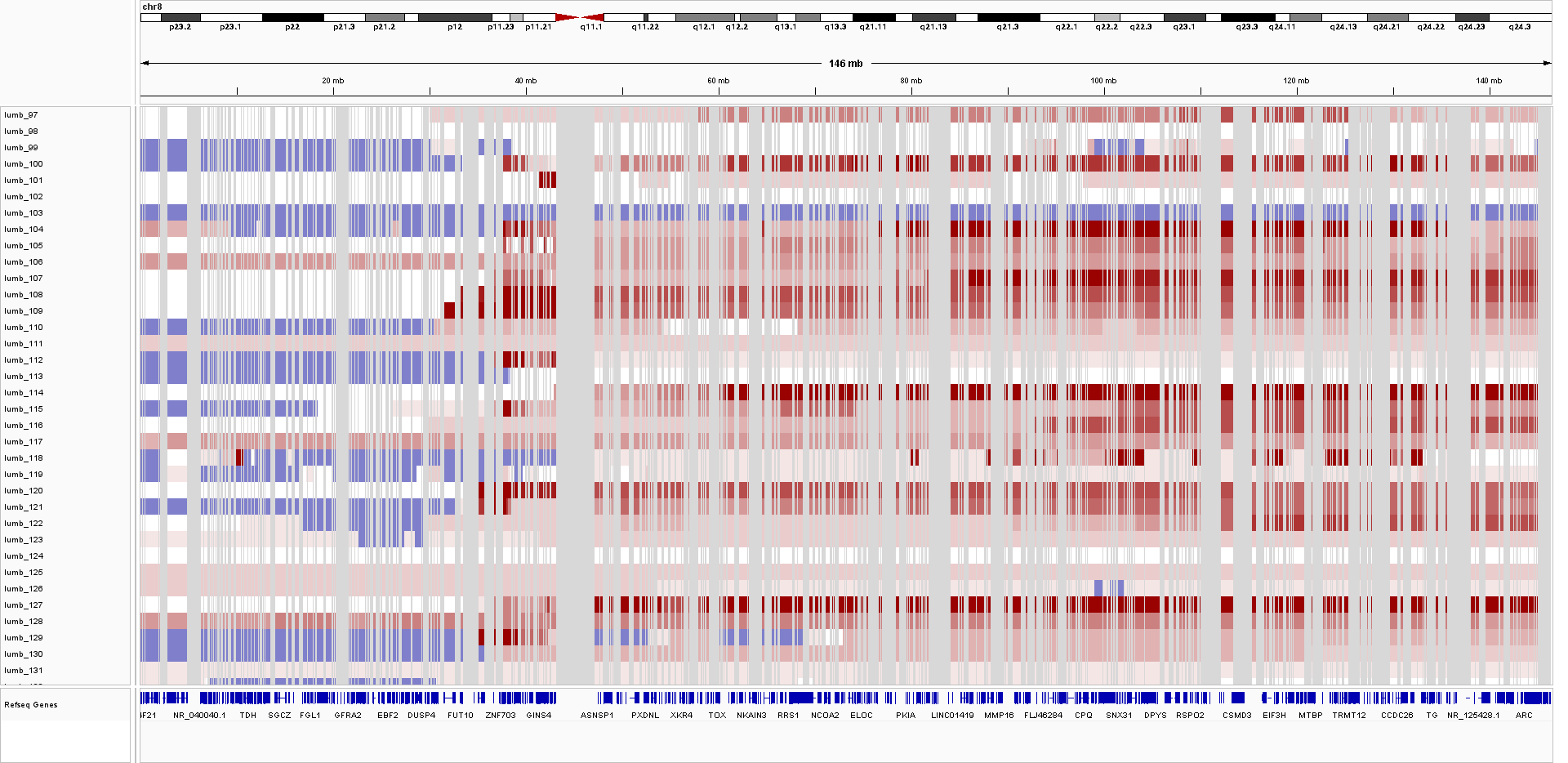

Supplement: Supplementary file 1 [file DataSheet1.zip › Supplementary/S7.png]

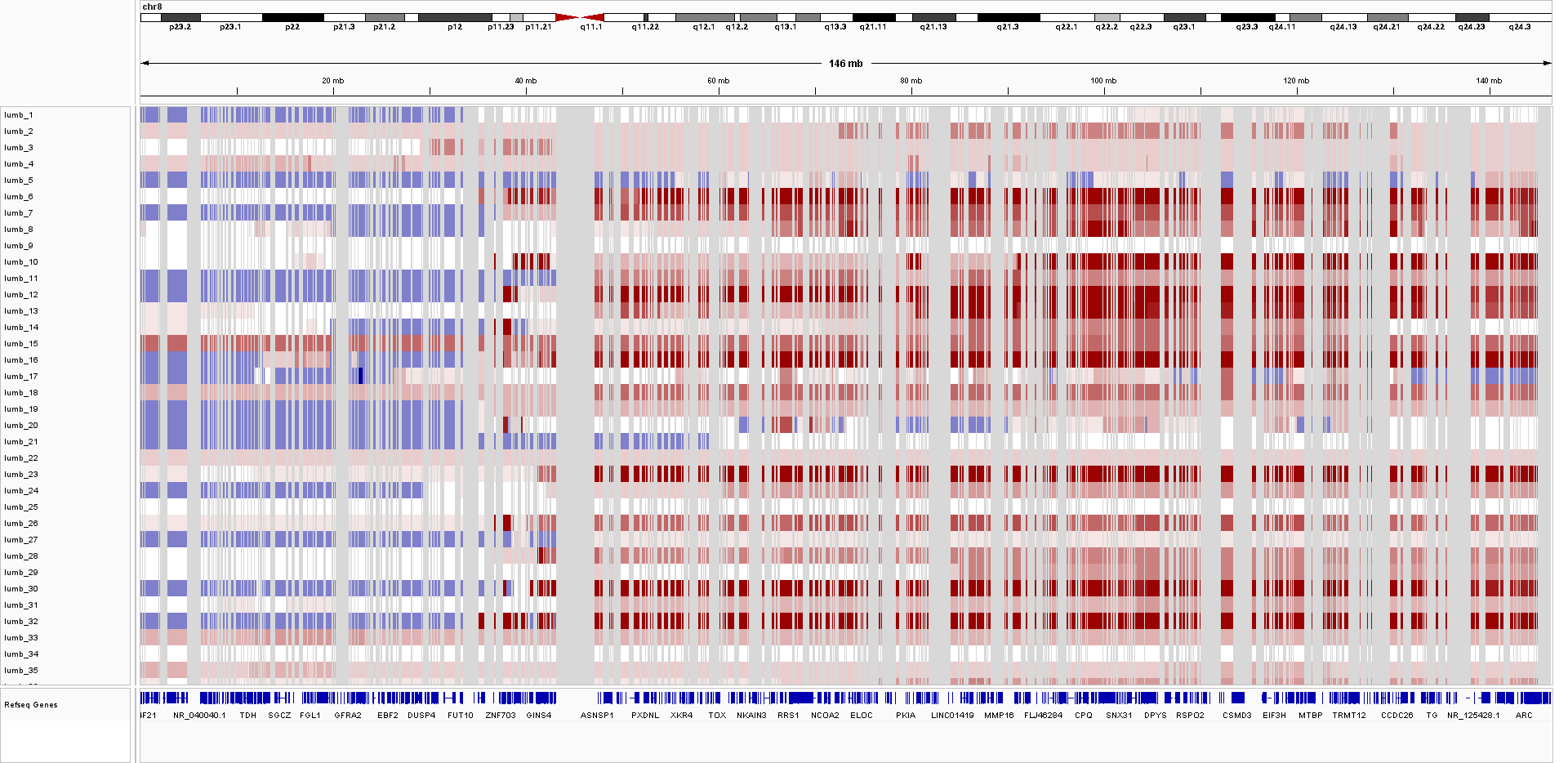

Supplement: Supplementary file 1 [file DataSheet1.zip › Supplementary/S5.png]

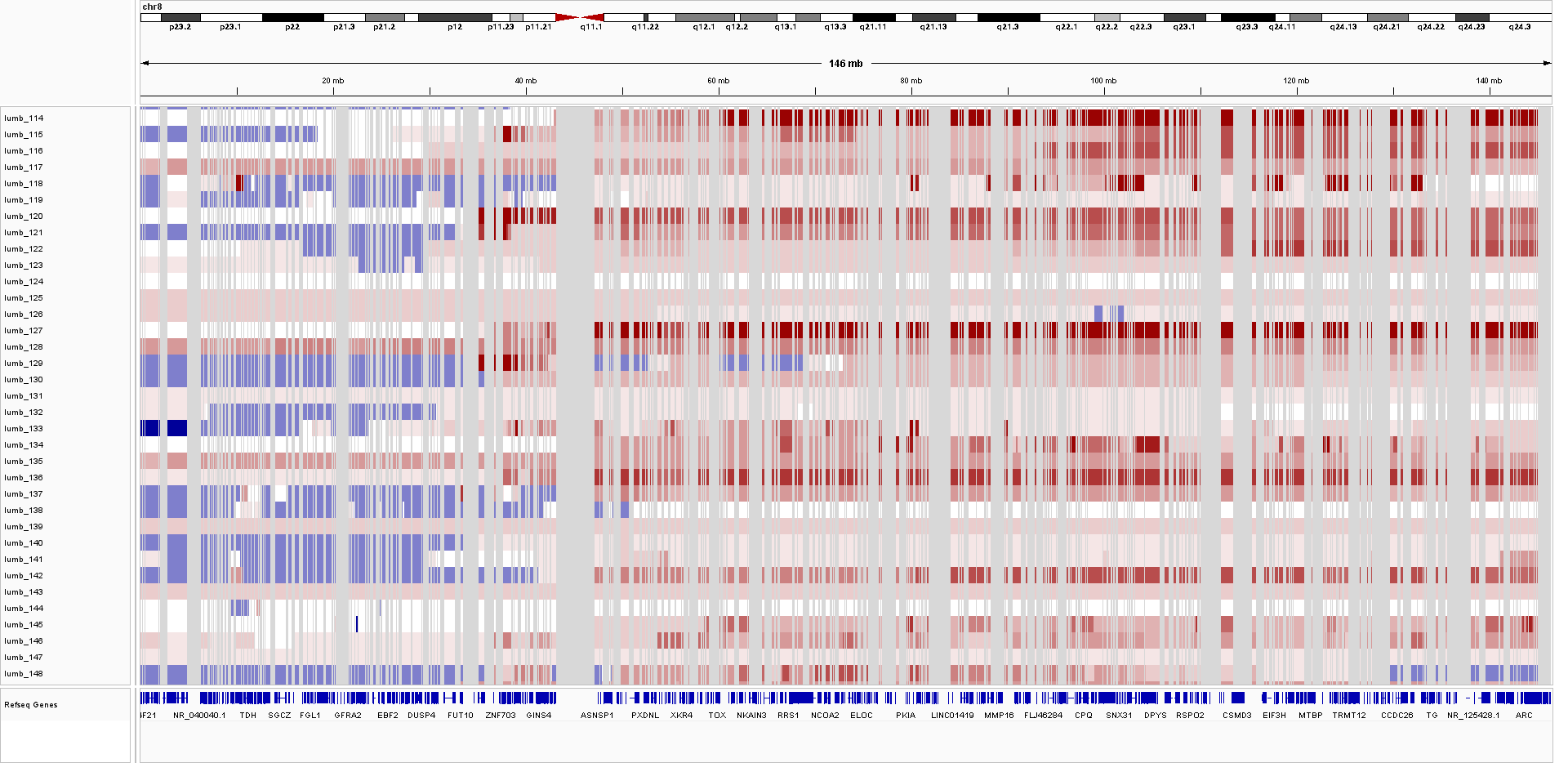

Supplement: Supplementary file 1 [file DataSheet1.zip › Supplementary/S8.png]

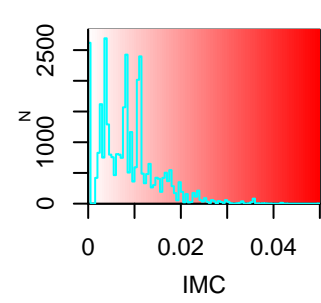

Heatmap Kolmogorov–Smirnov, statistic D

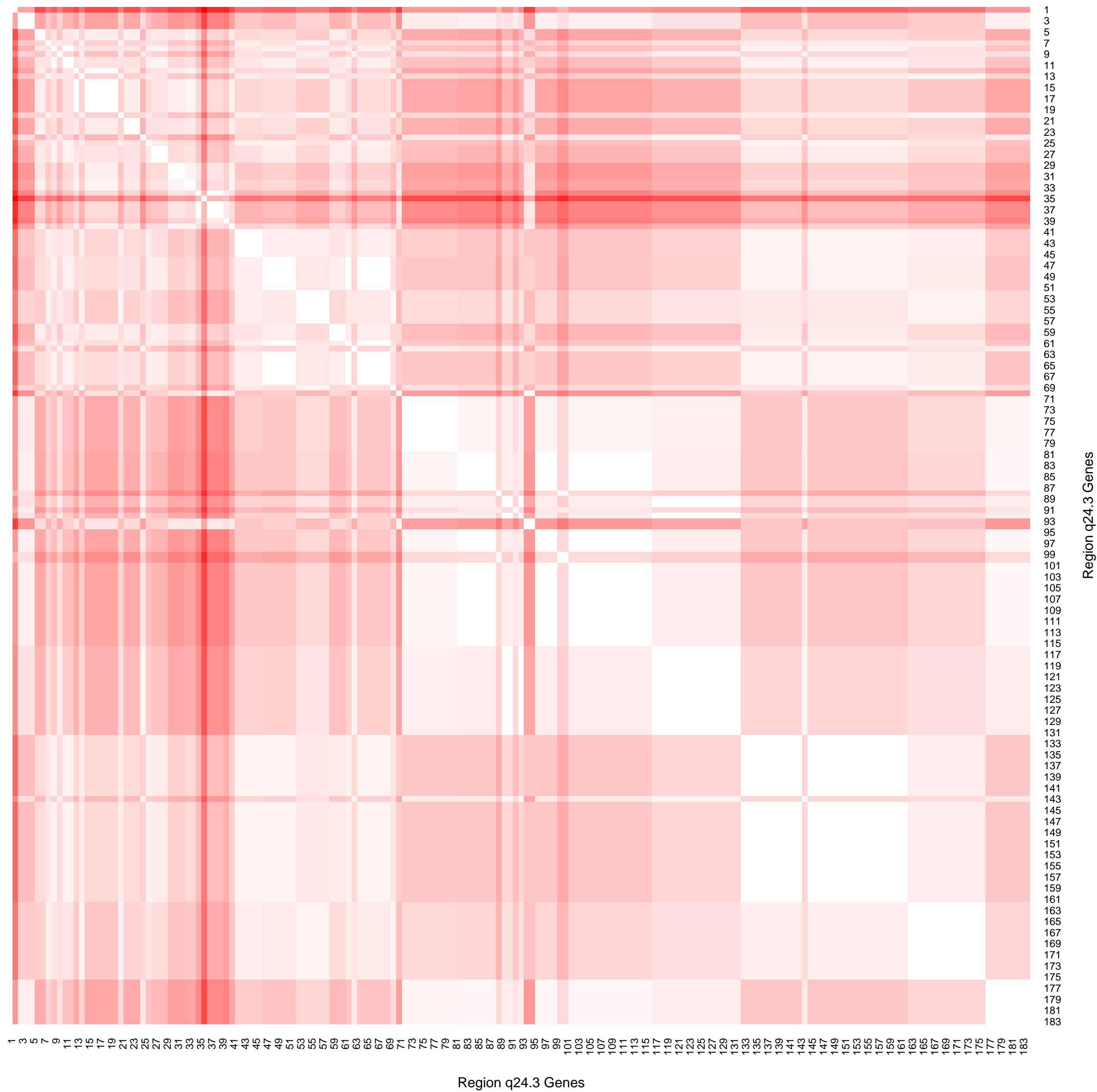

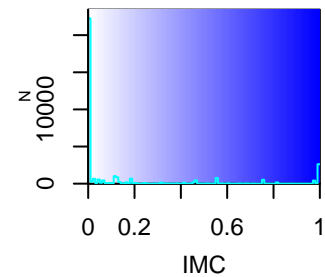

Heatmap Kolmogorov–Smirnov, p-values

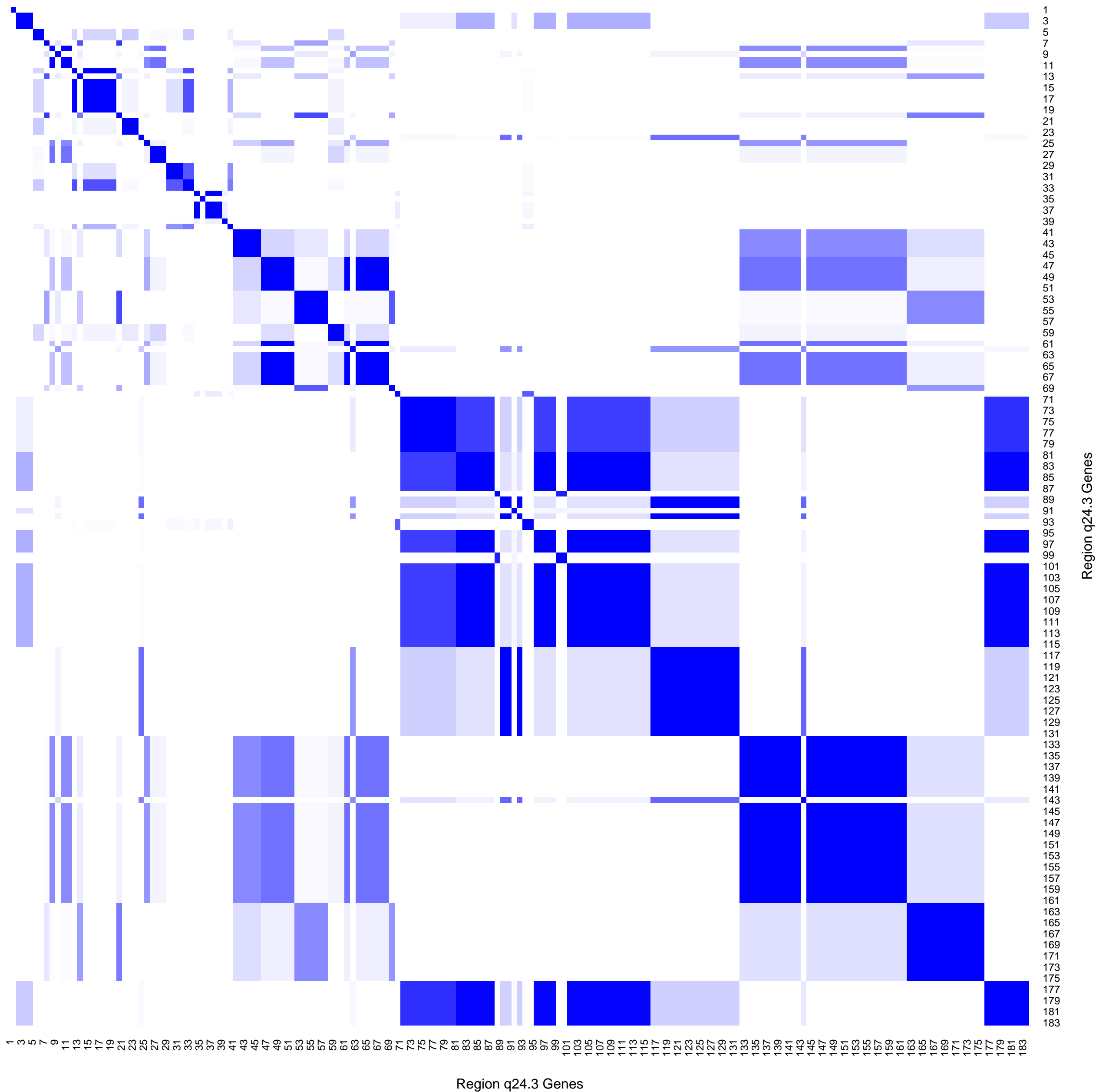

Supplement: Supplementary file 1 [file DataSheet1.zip › Supplementary/S9.pdf]
